# Randomized controlled pilot of an intervention to reduce and break-up overweight/obese adults’ overall sitting-time

**DOI:** 10.1186/s13063-015-1015-4

**Published:** 2015-11-02

**Authors:** Pedro B. Júdice, Marc T. Hamilton, Luís B. Sardinha, Analiza M. Silva

**Affiliations:** Universidade de Lisboa, Exercise and Health Laboratory, Interdisciplinary Center for the Study of Human Performance, Faculdade de Motricidade Humana, Estrada da Costa, 1499-002 Cruz-Quebrada, Portugal; Texas Obesity Research Center, Houston, TX USA

**Keywords:** Sedentary-time, Leisure-time, Reduction, Breaks, Physical activity, Workplace

## Abstract

**Background:**

Too much prolonged sitting is a prevalent health risk among adults. Interventions have focused mainly on the workplace, with limited attention to non-work settings. The effectiveness of a short-term intervention to reduce and break-up sitting-time in overweight/obese adults was examined. This pilot study sought to determine the feasibility of interrupting sitting to stand/ambulate objectively with ActivPAL devices which provide a valid measurement of sit/stand transitions.

**Methods:**

This is a cross-over randomized controlled pilot that included 10 participants (aged 37–65 years) and although a small and short-term intervention (1-week intervention; no washout) further informs on the feasibility of interventions on a larger scale. At the workplace, screen-delivered hourly alerts prompted participants to break-up sitting-time through adopting walking behaviors (approximately 30–60 minutes day^−1^). During transportation/home/leisure-time individual goals for steps day^−1^ were set and sitting-reduction strategies (including behavioral self-monitoring) were delivered through daily text messages. Change in inclinometer-derived sitting-time is the main outcome. Standing, stepping, number of sit/stand transitions and participant satisfaction were also examined.

**Results:**

For the intervention compared to the control-week (mean difference (95 % confidence interval); *p* value), participants had less sitting-time (1.85 hours (0.96–2.75); *p* = 0.001), more standing (0.77 hours (0.06–1.48); *p* = 0.036), and more stepping (1.09 hours (0.79– 1.38); *p <* 0.001). Importantly, there was no change in the total number of sit/stand transitions (3.28 (−2.33–8.89); *p* = 0.218) despite successfully reducing sitting-time and increasing time spent standing and walking.

**Conclusions:**

Sitting-time in overweight/obese adults can be reduced following a brief multi-component intervention based on prompts, telephone support, goal setting and behavioral self-monitoring. However, the results from this pilot study provide new insight that when overweight/obese adults attempted to reduce sedentary-time by walking and standing for approximately 2 hour day^−1^ more than usual, they did not actually get up from sitting more often (i.e. increasing the number of sit/stand transitions), but instead remained on their feet for longer during each non-sitting bout. This behavioral resistance to make more sit/stand transitions (i.e. get-up from sitting more often) may have important implications for future modification programs and supports the concept that when overweight/obese people are sitting, people seem to prefer not to interrupt the sedentary behavior to get-up from sitting.

**Trial registration:**

26 November 2013, ClinicalTrials.govID:NCT02007681 (first participant was randomized on 2 September 2013).

**Electronic supplementary material:**

The online version of this article (doi:10.1186/s13063-015-1015-4) contains supplementary material, which is available to authorized users.

## Background

Sedentary behavior – time spent sitting or reclining during waking-hours [1] is a specific occupational hazard in office workers. Prolonged sitting is associated with obesity [2], metabolic disorders [3] and all-cause mortality [4] and observational [5] and experimental evidence [6, 7] suggest that interrupting sitting-time may be associated with better health outcomes. Adults spend most of their time in sedentary behaviors, some 65 % of waking- hours; 8 to 11 hour day^−1^ [8] and one of the features of modern life is that work has become less physically active and more sedentary [9] and has more leisure-time engaged in sitting-related pursuits [10].

There is a lack of studies that aim to understand how the two desirable dimensions from these interventions (total sitting-time reductions and increases in sit/stand transitions) would in fact interact in real-life settings. The workplace is an important context to introduce strategies for reducing and breaking up periods of prolonged sitting [11]. However, leisure-time and non-working days also comprise a large portion of a working adult’s week [10]. Recent trials have shown significant reductions in workplace sedentary-time, using sit/stand workstations [11], educational sessions [12] and multi-component interventions [13]. These multi-component interventions are likely to provide the most effective approach to reduce workplace sedentary-time [14, 15]. In addition, interventions using prompts to disrupt sitting-time and increase physical activity (PA) at work have been shown to effectively increase the number of breaks in sitting-time and to reduce the number of bouts of prolonged sedentary-time [16–18]. Distinct prompt frequencies have been previously used [16–18] but regardless of a generalized increase in the number of breaks, shorter prompt frequencies (1 prompt every 30 minutes) seemed not to reduce overall sitting-time [16]. Therefore, in this pilot study we considered hourly prompts to enhance overall sitting-time reduction while at work.

Recent investigation has been shown that workers whot spend more time in sedentary pursuits during working hours do not compensate by being more active in non-working periods [19]. Prior interventions aiming to increase PA in employees have been found to be of benefit [20, 21]. To reduce overall daily sitting-time, there is a need for interventions that, in addition to focusing on the workplace context, also target leisure-time contexts [19].

Furthermore, a recent systematic review [24] showed that overweight/obese people are an understudied population group in interventions that target reductions in sitting-time [24] with only 2 studies [15, 25] including overweight/obese people (body mass index (BMI) > 25.0 kg m^−2^). In fact, those are the persons that are at higher risk of several diseases [26]; therefore, this pilot study tried to fill this gap by examining the short-term effectiveness of reducing and breaking up overall daily sitting-time of physically-inactive overweight/obese working adults using a multi-component intervention simultaneously addressing workplace and leisure-time contexts.

## Methods

For sample and power calculations (whether not reported here) we considered one of the main outcomes from this pilot study, the energy expenditure assessed by the doubly labeled water (DLW) technique. Based on a previous intervention [27], the energy expenditure assessed by DLW within each participant group was normally distributed with a standard deviation (SD) of 1.09. If the true difference in the experimental and control means was −1.31 MJ day^−1^, we would need to study 10 experimental participants to be able to reject the null hypothesis that the population means of the experimental and control were equal with probability (power) 0.8. The Type I error probability associated with this test of this null hypothesis is 0.05.

The study was approved by the Faculty of Human Kinetics, University of Lisbon Ethics Council (approval number: 14/2013) and conducted in accordance with the Declaration of Helsinki [28]. Written informed consent was obtained prior to entry into the trial. After a careful analysis of the work patterns of several academic and administrative sectors of the university and surrounded workplaces (*n* = 50), an invitation Email was sent to each potential workplace limiting the participation to 1 person per workplace to avoid behavioral coupling or contamination between participants. Therefore, an invitation Email was sent to 50 potential participants working full-time that involved prolonged computer-based work while sitting. Details of the study were explained to respondents (*n* = 30) via telephone calls and participants who expressed interest (*n* = 20) attended a 30-minute face-to-face screening session.

**Fig. 1:**
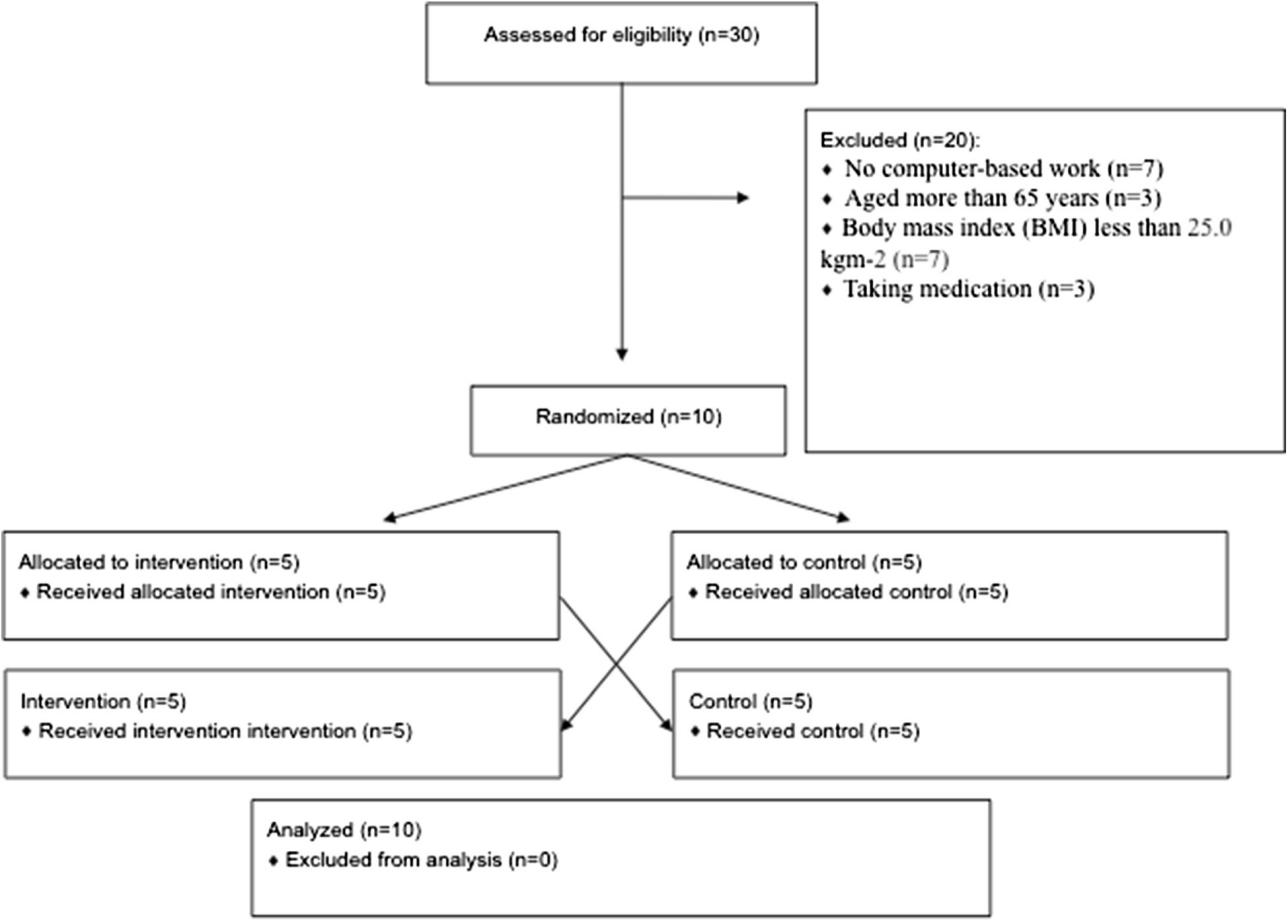
Screening, enrollment and interventions of the study participants

Inclusion criteria consisted of: being currently employed in a full-time academic or administrative role that involves greater than 7 hour day^−1^ computer-based work; 18–65 years old; BMI greater than 25.0 kg m^−2^; not taking any medication or dietary-supplements; being physically inactive (not meeting the moderate-to-vigorous PA (MVPA) recommendations and with approximately 5000 steps day^−1^); and being free from any major disease that would inhibit their ability to participate in the study. Based on eligibility criteria we tested ten participants (five women and five men) (Fig. 1). Considering the ten participants’ occupation, five had an administrative role in five different departments of the university. Two participants worked in a bank (distinct banks). One was a lawyer working in a private company and the last two participants were independent architects working in their own private studios. There was no drop-out during the trial.

The pilot study design consisted on a cross-over randomized clinical trial (ClinicalTrials.govID: NCT02007681). A baseline measurement period (1 week), followed later (with approximately a 1-week break) by a 2-week period of measurement, where one week was the intervention and one the control (with the order of intervention and control randomized by an automated computer-generated randomization scheme). Data were collected between September and December 2013 and analyzed in 2014 Additional files 1 and 2.

To ensure that participants were physically inactive (< 30 minutes day^−1^ of MVPA and approximately 5000 steps day^−1^) and to assess habitual steps day^−1^, PA and sedentary-time, participants were fitted with an accelerometer (Actigraph GT3X+, Actigraph, Pensacola, FL, USA) prior to the intervention. The pilot trial consisted of two 1-week conditions performed in a random order, both under free-living conditions: intervention (asked to make a 3-hour reduction in sitting-time) and control (asked to undertake habitual sitting-time). In each condition participants were instructed to maintain the same eating patterns while wearing an accelerometer (Actigraph GT3X+, Actigraph, Pensacola, FL, USA) and an inclinometer (ActivPAL, PAL Technologies Ltd., Glasgow, UK). Both these devices do not allow participants to have a real-time feedback on their PA levels. Therefore, a pedometer (OMRON, Walking style II, HEALTHCARE Ltd., Kyoto, Japan) was used on the right hip, near the iliac crest, during waking-hours, and the participants were requested to remove it only during water-based activities such as showering and swimming, so that they could control the number of steps they were performing during the day.

Regardless of the randomization order, participants were told verbally in person to maintain their habitual PA levels and sedentary patterns in both working hours and non-working periods during the control week. We reinforced to them the importance of never exceeding the daily step goal for this condition (the number of steps day^−1^ performed at baseline) by using telephone calls and text messages throughout the day as well as checking on adherence to these individual daily steps goal, and reminding participants to report their daily steps in a diary.

To avoid carryover of behavioral strategies to reduce sitting-time adopted during the intervention week, those participants who were randomly allocated to the intervention condition during the first week were explicitly instructed to follow their normal work and non-work routines on the first day of the control week. Furthermore, on the last day of the intervention week, an investigator met the participants and explained to them verbally in person that starting on the next day (beginning of control week) it was critical that they did not perform any efforts to change their habitual activity patterns (prior to the study starting).

The intervention at the workplace to reduce sitting-time included a software program (Workrave, GitHub) that gave hourly alerts to participants to break-up their sitting-time for approximately 7 minutes through taking part in walking (to accumulate 30–60 minutes day^−1^). This software was installed in the work computers and automatically alerted the participants to break-up working while seated by presenting a warning message that covered and locked the screen entirely. When this alert appeared in the screen, participants had the option to postpone for 5 minutes, but the second time they did not have any option but to stop working and perform a break for at least 7 minutes (the time during which the computer screen was locked).

During transportation/home/domestic/leisure-time contexts, individual goals for number of steps day^−1^ were set based on an expected step cadence for ambulatory activities (approximately 90–120 minutes day^−1^) and by adding 6000 steps to their initial habitual daily amount. Also, generic strategies to reduce and break-up sitting-time were suggested, and participants identified strategies specific to their circumstances in their work, transport, and home contexts, for attaining their goal (3-hour-reduction in sitting-time).

Daily adherence during the intervention week was managed by using motivational telephonecalls and text messages throughout the day as well as checking on adherence to the individual daily steps goal, and reminding participants to report their daily steps in a diary, which also worked as part of the intervention.

Participant satisfaction with the program was rated during the post-intervention assessment on a scale of 1–10, with 1 being extremely displeased and 10 being extremely satisfied. They also had to select one of the three strategies (screen-based prompts; daily steps goal; and behavioral strategies personally delivered) and one from the two domains (work/leisure-time) as the most effective for reducing sitting-time.

Anthropometric variables were measured according to the standardized procedures described elsewhere [29]. BMI was calculated as body mass (kg) height^−*2*^ (m).

ActivPAL Professional (PAL Technologies Ltd., Glasgow, UK) monitor was considered the primary method for the main variables in this study, as it provides a reliable method for differentiating sitting/lying, standing and stepping activities [30], with a high accuracy for time spent sitting, compared with direct observation [31]. The ActivPAL is a uniaxial piezoresistive accelerometer and inclinometer which is small (35 mm × 53 mm × 7 mm) and lightweight (20 g) worn on the middle-anterior line of the right thigh and provides a variety of objectively measured and objectively processed variables, including total time spent sitting/lying, standing and stepping and sit/stand transitions. Data were collected at a predetermined 10 Hz and the 15-second interval output was used for data analysis. Recorded output from the ActivPAL monitor was downloaded, processed, and classified into sitting, standing, and walking by using manufacturer-supplied ActivPAL software (version 5.9.1.1, PAL Technologies Ltd., Glasgow, UK).

From the 15-second interval output it was possible to extract prolonged and uninterrupted periods of time spent sitting, standing and stepping of different durations (bouts of ≤ 4 minutes; 5–9; 10–19; 20–29; 30–59; and at least 60 minutes) by manually counting the number of bouts in which participants were sitting, standing or stepping in the bout’s duration categories [32]. Because the past 3 bout categories are infrequent for the standing bouts (20–29; 30–59; and at least 60 minutes), they were combined into 1 category (≥ 20 minutes).

The device was attached to the skin with a manufacturer-supplied non-allergenic and non-waterproof adhesive tape (PALstickie, PAL Technologies Ltd., Glasgow, UK) and used continuously for 24 hours a day for 14 days, except for water-based activities such as showering and bathing. After showering or bathing participants were instructed to re-attach ActivPAL with an additional piece of the same adhesive tape that we provided. Prior to the trial we taught them exactly how, where and the correct positioning to attach the device. None of the participants performed any activity like swimming or any other water-based activity. Therefore, a valid-day was defined as having ≥ 22 hours of monitor wear, corresponding to the minimum daily use except for the showering and bathing. Participants were asked to record waking/sleeping hours and wear-time in a logbook. The information provided in the diary was used to determine ActivPAL’s waking period and, therefore, to assess sedentary-time between waking and bed times. All ActivPAL’s main variables including the sit/stand transitions, and the number of bouts in which participants were sitting, standing or stepping do not include sleeping hours. They were asked to record timing and reasons for every occasion the ActivPAL was removed.

Additionally, all participants were asked to wear an accelerometer Actigraph GT3X+ (Actigraph, Pensacola, FL, USA) on the right hip, near the iliac crest, during waking-hours, and requested to remove it only during water-based activities such as showering and bathing [33]. The device activation, download, and processing were performed using the software Actilife (v.6.9.1, Actigraph, Pensacola, FL, USA). A valid-day was defined as having 600 or more minutes (≥ 10 hours) of monitor wear, corresponding to the minimum daily use of the accelerometer [34]. As well as reported monitor non-wear-time (i.e. when it was removed for sleeping or water activities), periods of at least 60 consecutive minutes of zero activity intensity counts were also considered as non-wear-time [35].

The amount of activity assessed by the Actigraph accelerometer was expressed as minutes per day spent in different intensities. The cutoff values used to define the intensity of PA and, therefore, to quantify the mean time in each intensity (sedentary, light, moderate or vigorous) were: sedentary: < 100 counts min^−1^; light: 100–2019 counts min^−1^; moderate: 2020–5998 counts min^−1^ (corresponding to 3–5.9 metabolic equivalents (METs); vigorous: ≥ 5999 counts min^−1^ (corresponding to ≥ 6 METs) [36]. There are no cutoffs for the sedentary-time using the 3-axial information from this new generation Actigraph GT3X+ accelerometer; therefore, we used the previous cutoffs which utilize the vertical axis only. Actigraph break was considered as any bout of time in which the accelerometer count rose up to or above 100 counts min^−1^ and which stayed within the light-intensity physical activity (LIPA) interval (< 2020 counts min − ^1^).

The delivery and fitting of both devices (ActivPAL and Actigraph GT3X+) to the participants were conducted face-to-face [34]. The devices were activated on the first day at 6.00 am and data were recorded in 15-second epochs. Participants were asked to record timing and reasons for every occasion the devices were removed. Although it would be useful to differentiate working from leisure-time periods, participants were not told to record the times they entered and finished work.

The primary outcomes were: ActivPAL’s total waking-time spent sitting, standing, stepping; number of steps; and the number of bouts (≤ 4 minutes; 5–9; 10–19; 20–29; 30–59; and at least 60 minutes) of uninterrupted sitting. As secondary outcome measures the number of bouts (≤ 4 minutes; 5–9; 10–19; 20–29; 30–59; and at least 60 minutes) of ActivPAL’s standing and stepping and the Actigraph accelerometer’s breaks in sedentary-time were considered.

Energy and nutrient intake were assessed in 3-days (1 weekend-day) in each condition week, using 24-hour diet records. Participants were instructed regarding portion sizes, supplements, food preparation aspects (boiling, grilled, frying), and others aspects (e.g. fried in olive oil or butter) pertaining to an accurate recording of their energy intake. At the last visit, records were turned in and reviewed for liquid ingestion, macro-nutrient composition and total energy intake by the same technician. Diet records were analyzed using Elizabeth Stewart Hands and Associates (ESHA’s) Food Processor Nutrition Analysis software for Windows version 10.0, 2013 (SQL Inc., an ESHA Company, Salem, OR, USA).

Statistical analysis was performed using PASW Statistics for Windows version 21.0, 2010 (SPSS Inc., an IBM Company, Chicago, IL, USA). Descriptive analysis included means and SD for all measured variables. Changes in the main primary and secondary variables between control and intervention conditions and for week-days with weekend-days were individually assessed using paired sample *t* tests. Day-by-day variations in sitting-time and treatment by condition interactions were examined by repeated measures analysis of variance (ANOVA). The distributional assumptions for ANOVA are for the normal distribution of the residuals. Therefore, normality was found for the residuals from all the main variables. To test if the randomly assigned order of treatment or the treatment by group interaction influenced the differences between conditions, the order of randomization was entered as between-subject variable and interaction with the main variables’ changes were checked. Statistical significance was set at 0.05.

## Results

All participants completed both trial conditions and no adverse events were reported. Of the ten participants (five women; five men), two were overweight and eight were obese. Mean age was 50.4 (SD = 11.5; min–max = 37–65) years; mean BMI was 32.6 kg m^−2^ (SD = 5.50; min–max = 25–41). Actigraph measured daily mean sedentary-time at baseline was 688 (SD = 91.2; min–max = 565–846) minutes; mean LIPA was 170 (SD = 25.4; min–max = 130–193) minutes; MVPA was 28.1 (SD = 12.4; min–max = 8–27) minutes; and the daily mean number of steps was 4783 (SD = 1365; min–max = 1274–5803).

For both intervention and control weeks there were no differences between week-days and weekend-days for any of the ActivPAL variables; therefore, week-days and weekend-days were pooled together. Also, no differences were found for the dietary patterns between conditions, (mean, SD control; mean, SD for the intervention-control, *p* value); energy intake (1828, 635 Kcal; −105, 439 Kcal, 0.468), carbohydrates (239, 169 g; −36.2, 134 g, 0.416), fat (63.9, 22.9 g; −5.67, 20.8 g, 0.533), and protein (78.3, 26.2 g; −1.93, 25.8 g, 0.818).

Daily overall waking-time during the control week was 16.4 hour day^−1^ and 17.0 hour day^−1^ for the intervention week. Individually, reductions in waking-hours sitting-time varied from 4.8 % (0.56 hour day^−1^) to 36 % (4.16 hour day^−1^), standing-time varied from 1.0 % reduction (−0.05 hour day^−1^) to 62 % increase (2.71 hour day^−1^), and stepping-time increased from 41 % (0.51 hour day^−1^) to 145 % (1.80 hour day^−1^). Sitting-time in 2 participants was reduced more than the target of 3 hour day^−1^ reductions, 6 reduced sitting more than 1 hour day^−1^ and 2 achieved a reduction in sitting-time of less than 1 hour day^−1^.

As presented in Table 1, for the intervention week compared to the control week, there were significantly fewer daily hours spent sitting and there was significantly more time spent standing, stepping and a greater number of daily steps. There were no significant differences in ActivPAL-determined daily sit/stand transitions. Because the number of sit/stand transitions was not reduced, most commonly the additional standing and walking bouts were occurring continuously (i.e. slightly longer non-sitting bouts for the cumulative duration of approximately 1.85 hour day^−1^). This resulted in greater number of bouts of ≤ 4 minutes of standing and ≤ 4-minute and 5–9-minute bouts of stepping (Table 1). There were no significant differences between conditions for any of the sitting bouts, standing bouts longer than 5 minutes, stepping bouts longer than 10 minutes and Actigraph breaks as defined by > 100 counts min^−1^. Neither the randomly assigned order of treatment nor the treatment by groups’ interaction had any statistically-significant effect on these differences (*p* > 0.05). The (mean, SD) for the overall sitting-time in the control and intervention conditions was 11.99, 1.19 and 10.23, 1.64 hour day^−1^, respectively in the group that started in the control condition followed by the intervention. The participants who performed the 2 conditions in an inverse order (intervention first and control afterwards) spent 10.82, 1.51 hour day^−1^ of sitting-time during the control condition and 8.82, 2.19 hour day^−1^ in the intervention period.

**Table 1:** Differences in sitting, standing, stepping, daily steps, and bouts of sitting, standing, stepping from different durations (*n* = 10)

	Intervention	Control	Intervention minus Control difference	
	(Mean, SD)	(Mean, SD)	(Mean, SD)	*p*
During overall waking-time				
Sitting-time (hour day^−1^)	9.55, 1.80	11.40, 1.48	−1.85, 1.25	0.001
Standing-time (hour day^−1^)	5.16, 1.82	4.39, 1.40	0.77, 0.99	0.036
Stepping-time (hour day^−1^)	2.33, 0.37	1.24, 0.29	1.09, 0.41	<0.001
Sit/stand transitions (number day^−1^)	56.90, 9.06	53.60, 11.00	3.28, 7.84	0.218
Steps (number day^−1^)	12,076, 1934	5712, 1335	6363, 1953	<0.001
Sitting ≤ 4 minute bouts (number day^−1^)	31.20, 8.74	26.40, 10.80	4.83, 9.68	0.149
Sitting 5–9 minute bouts (number day^−1^)	9.60, 2.67	7.92, 2.46	1.68, 2.79	0.088
Sitting 10–19 minute bouts (number day^−1^)	9.58, 3.07	7.62, 1.00	1.96, 3.00	0.069
Sitting 20–29 minute bouts (number day^−1^)	4.31, 1.55	3.81, 1.24	0.50, 1.50	0.320
Sitting 30–59 minute bouts (number day^−1^)	4.09, 1.86	3.95, 1.03	0.14, 1.78	0.805
Sitting ≥ 60 minute bouts (number day^−1^)	3.13, 1.46	3.33, 1.36	−0.21, 1.03	0.542
Standing ≤ 4 minute bouts (number day^−1^)	491.00, 88.50	365.00, 78.50	125.00, 104.00	0.004
Standing 5–9 minute bouts (number day^−1^)	5.56, 2.92	5.55, 2.44	0.01, 2.51	0.995
Standing 10–19 minute bouts (number day^−1^)	1.29, 0.84	1.49, 1.37	−0.21 , 0.89	0.480
Standing ≥ 20 minute bouts (number day^−1^)	1.70, 2.12	2.10, 3.96	−0.40, 3.78	0.745
Stepping ≤ 4 minute bouts (number day^−1^)	19.10, 6.67	3.00, 2.71	16.10, 6.95	<0.001
Stepping 5–9 minute bouts (number day^−1^)^a^	2.94, 1.03	0.46, 0.42	2.48, 1.07	<0.001
Actigraph breaks (100 counts min^−1^ threshold, number day^−1^)	506.00, 106.00	477.00, 128.00	29.10, 7.50	0.085

*SD* standard deviation

^a^The participants had no stepping bouts longer than 10 uninterrupted minutes on both control and intervention weeks. Therefore, the means and differences were not presented for these bouts lengths, as it would be all zero.

All variables were obtained with ActivPAL except for Actigraph breaks, which were obtained with Actigraph GT3X+

**Fig. 2:**
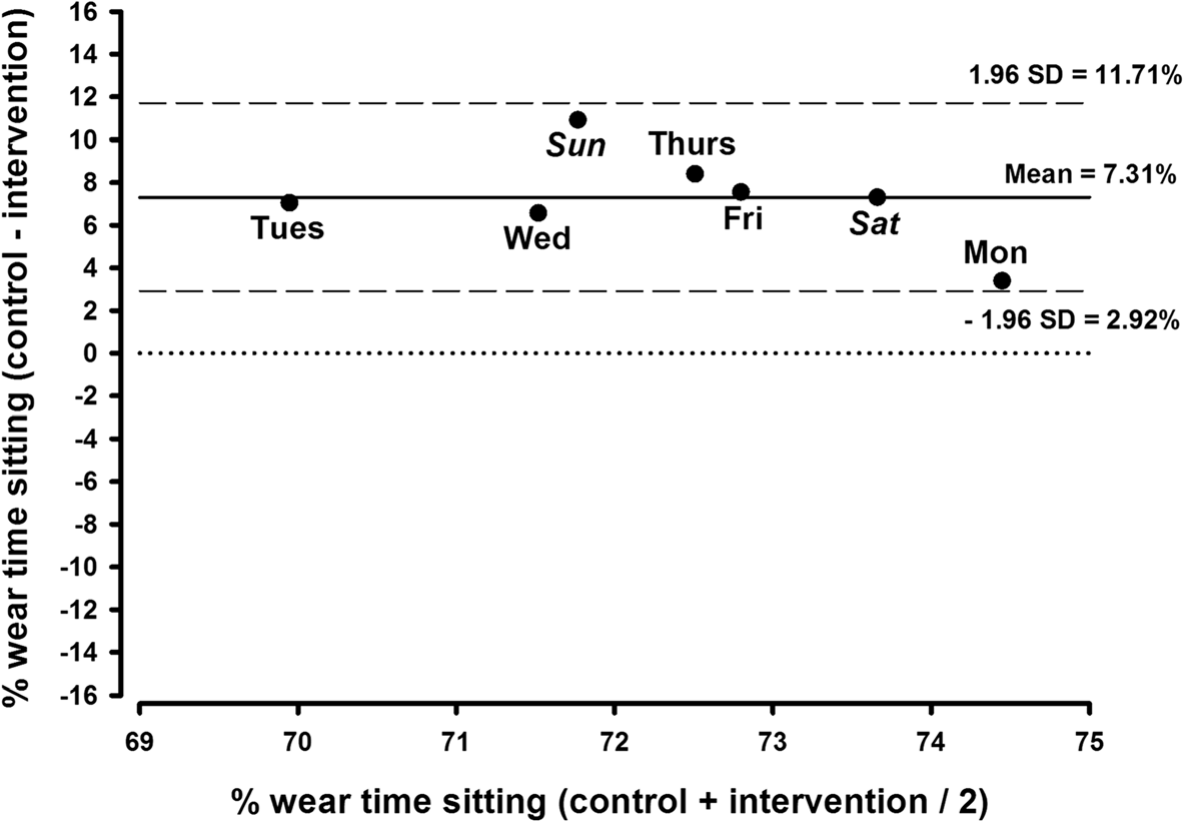
Percentage of sitting from wear-time within the trial: a Bland-Altman analysis for the differences between control and intervention days

No significant day-by-day variations in sitting-time (*p* > 0.05) were observed in either the control or intervention week (Fig. 2).

On a scale of 1–10, with 1 being extremely displeased and 10 being extremely satisfied, 6 of the 10 participants rated the program 10, (median = 9.5, min–max: 8–10). The ten participants rated the daily steps goal as the best strategy to achieve the sitting-time reduction and seven reported leisure-time to be the greatest domain to perform sitting-time changes.

## Discussion

The results from this pilot intervention showed a reduction of 110 minutes day^−1^ of sitting-time in overweight/obese adults following a brief multi-component intervention based on prompts, telephone support, goal setting and behavioral self-monitoring. Along with the findings from previous interventions [17, 37] on the efficacy for shifting sedentary-time by walking and standing for approximately 2 hour day^−1^, we extended these results by finding that participants did not actually get up from sitting more often. Although a small and short-term intervention, this pilot study offers valuable insight for the rapidly growing field of research aiming at reducing sedentary-time and increasing the number of activity breaks throughout the whole day. Using the most validated device [30, 31, 38] for measuring sit/stand transitions (ActivPAL), we found that people in the “real world” did not get up from sitting many times per day for activity breaks.

Previous interventions that also aimed to interrupt sitting-time have been effective in increasing the number of daily sit/stand transitions [16, 18] but the concomitant overall reduction in sitting-time was not always verified [16]. Therefore, it is not yet clear how the various metrics of number and total duration of sitting-time should be interpreted. However, only the numbers of sitting bouts within particular intervals of bout duration were reported in the present study and not the mean time in each of these bout-length categories. While this gives a reasonable cross-sectional description of the pattern of sitting, it is less useful at identifying the sorts of changes in the duration of sitting bouts falling within the intervals selected. For example, a reduction of a single sitting bout from 58 minutes to 31 minutes would be a reduction in total sitting time of 27 minutes, but this would result in no change in the number of sitting bouts in the 30–59-minute interval. Therefore, the sedentary-bout categories and the selected intervals must be considered a limitation.

From our interviews with participants and the ActivPAL data regarding stepping, it appears that much of the extra non-sitting-time was spent when participants went out on a slow walk. Examples of behaviors where people would perform slow and intermittent stepping and standing include more time shopping, cooking, and light non-exercise strolls in leisure-time. This behavioral resistance to make more sit/stand transitions in the present study (from 54 day^−1^ to 57 day^−1^ from before to during the intervention) and other interventions [39] (i.e. get up from sitting more often) would be consistent with a potentially important new concept regarding human sedentary behaviors: namely, that once people are engaged in a seated activity such as using the computer, reading a book, watching a movie, etc., people do not want to be interrupted to perform activity even if it is potentially healthy for them [24].

Reflecting on the habitual number of  > 60-minute sitting bouts found in these workers (approximately 3 bouts day^−1^) and the hourly prompts for breaking up sitting-time it would be expected that participants did not increased the number of sit/stand transitions to a greater extent. The mean number of sit/stand transitions based on ActivPAL was about 55 day^−1^ and participants did not take enough breaks to do their standing and walking to result in any detectable change (3.3 sit/stand transitions) in this pattern of sitting and non-sitting-time. Regardless of the non-statistical differences, a similar magnitude of change in sit/stand transitions was found to be significant in a previous study [40]. Therefore, it is curious that considering the mean daily number of  > 60 minutes sitting bouts in the present study (approximately 3 bouts day^−1^), 3 sit/stand transitions would be sufficient to interrupt these periods of prolonged sitting-time (> 60 minutes). Nevertheless, and acknowledging this is a pilot study, the finding that the number of sit/stand transitions did not increase during this pilot intervention may be valuable for informing the many upcoming sedentary behavior interventions, aiming at evaluating health outcomes, to be more cautious about assuming that less total daily sitting-time will be effectively spread throughout the whole day as desired.

Moreover, the 16.2 % reduction in overall sitting-time was 4 times greater than for previously reported sitting-reduction interventions in overweight/obese adults using treadmill working desks [41] or by using a lock-out device to reduce TV viewing-time (3.8 %) [42]. We acknowledge that the differences between our findings and these studies might be due to the fact that those studies considered a longer intervention period but also because the present study considered both work and leisure-time settings [18]. Studies have been using ActivPAL in sitting-reduction interventions [15], which is a more valid measurement for distinguishing sitting from LIPA [43]. However, in contrast to a workplace intervention that included both normal weight and overweight/obese adults, and which also used ActivPAL devices, a multi-component intervention, and reduced sitting-time by 89 minutes/8-hour workday and 33 minutes in the workstations-only group [44], the present results (110 minutes day^−1^) show a major reduction in overall daily sitting-time, suggesting that focusing not only on workplace but also considering strategies to reduce sitting-time in non-work settings may enhance the effectiveness of these interventions [18]. Another study that considered work, commute and leisure-time was able to reduce muscle inactivity time by 33 minutes day^−1^ using a simple tailored counseling in both normal weight and overweight/obese adults [45]. Therefore, the multiuse of different strategies to reduce sitting-time in the present pilot study seemed to improve the efficacy of these sitting-reduction interventions on reducing total sitting-time but not to increase the number of activity breaks throughout the day.

Considering leisure-time only interventions, based on the actual daily step change (approximately 6000 steps) we anticipate that the inclusion of a higher daily step goal than has been purposed (1500–2000 steps day^−1^) [12] would result in higher stepping-times, which could indirectly contribute for reducing sitting-time [22, 23]. The number of breaks in sedentary-time showed no significant differences between the 2 conditions (0.60 breaks sedentary hour^−1^) but Actigraph measurement of breaks is not a measurement of sitting to standing transitions but rather it is the transition from being motionless to moving, which also occurs during standing. Thus, the large number of breaks as measured by acceleration is a metric of change in movement rather than posture. Regardless, our findings were similar to a previous study (0.64 breaks sedentary hour^−1^, *p* = 0.005) including overweight/obese adults [46] which reinforces the idea that people are resistant to increasing the number of breaks in sitting-time even though significantly reducing total time spent sitting. There were also no differences in the number of prolonged sitting bouts of any duration between the 2 weeks, but the absence of these differences may be explained by a shortening of the sedentary bouts within the duration categories (e.g. a change from 58 to 31 minutes in prolonged sitting-time) as opposed to across them (e.g. a change from 31 to 29 minutes). Likewise, as with the example presented above, similar cases would justify the significant reduction in total sitting-time without a corresponding change in the number of prolonged sitting bouts. However, the number of ≤ 4-minute bouts of standing and ≤ 4-minute and 5–9-minute bouts of stepping were significantly higher in the intervention week compared to the control week. In fact, while there are hundreds of standing bouts, there are only 50–60 sitting bouts, which may indicate that standing bouts are broken predominantly by stepping bouts (usually < 1-minute step bouts).

The lack of significant differences for the sitting bouts may also be related to the large variability concerning free-living conditions and the small sample size. In fact, this study was not powered for this hypothesis, representing a limitation. Furthermore, the fact that our daily step goal was higher than in previous interventions may justify the need for participants to perform longer walking bouts and consequentially they had fewer opportunities to break-up sitting-time. Methods to induce more breaks in sedentary-time over the whole day are challenging in free-living conditions and the present pilot study shows that it will be harder than expected to change this than simply getting people to go on one or two longer strolls/standing bouts.

The non-existence of a washout period between the two conditions could be considered a fair limitation to this study given that it is a lifestyle intervention, and some behavioral carryover might exist for the group that started with the intervention condition, and then participants would continue the intervention regardless of being in the control group. This possible response would lower the difference in sitting-time between the two conditions. However, neither the randomly assigned order of treatment nor the treatment by groups’ interaction had any statistically significant effect on these differences and no carryover existed; in fact, the group that started with the intervention condition was the one presenting higher differences between control and intervention conditions. Regardless, there was a trend for this group to present lower overall (control and intervention) sitting-time compared to the group that started with the control condition.

The lack of a good measure to distinguish work-time from leisure-time makes it difficult to objectively understand in which domain the major changes in sitting-time occurred and also what strategy from this multi-component intervention was more successful. These are some limitations that future interventional studies should be aware of. Regardless, based on participants’ choices, the daily step goals, the most easily understood strategy for reducing sitting-time and leisure-time, was the setting in which seven in ten reported as being the easiest to reduce sitting-time. The small sample size and the short-term duration of the trial are probably the main limitations of this pilot study. However, the results are important for guiding this rapidly emerging field because we found, with the most valid measurement tools for sitting-time and breaks from sitting, that even when making moderate reductions in overall sitting-time of almost 2 hour day^−1^, overweight/obese people did not get up from sitting more frequently than normal. While small efficacy studies could obviously use prompts (“alarms,” text messages, or other reminders) throughout the day to get up from sitting, it is perhaps important for scalable behavioral change in large numbers to carefully design behavioral studies that recognize that getting up from sitting when engaged in most tasks may be the hardest measure of sedentary behavior to make long-term changes in.

Strengths of our pilot intervention include the cross-over randomized controlled trial design, the focus on overweight/obese adults (who are an understudied population group in interventions that target reductions in sitting-time) the use of a multi-component intervention that extended strategies to the non-work settings, and also the use of objective and accurate measures of sitting-time (ActivPAL) [30, 31, 38]. Non-work days and leisure-time activities like TV viewing-time or computer screen-time also contribute to overall sedentary profile [42] and the present study adds to the scientific findings by taking a broader approach to influencing overall sitting-time. Also, a strength of this study was the fact that changes in dietary patterns were monitored and no differences were observed between conditions, meaning that participants did not increased their food intake in response to a higher activity level.

## Conclusions

The results from this pilot study suggest that a multi-component intervention focusing not only on the work environment but also on the reduction of sitting-time throughout the whole day may result in greater changes than single-context interventions. The magnitude of sitting-time changes in this pilot study, along with the poor increases in the number of sit/stand transitions and the utilization of objective measures, justify future investigations aiming to replicate the present approach on a larger scale and to understand if most effective “real-world” interventions are going to be found easily.

## Additional files

Additional file 1:
**CONSORT 2010 checklist of information to include when reporting a randomized trial*.** (DOC 217 kb) Additional file 2:
**CONSORT 2010 flow diagram.** (DOC 48 kb)

## Abbreviations

ANOVA: analysis of variance
BMI: body mass index
CONSORT: Consolidated Standards of Reporting Trials
DLW: doubly labeled water
ESHA: Elizabeth Stewart Hands and Associates
LIPA: light-intensity physical activity
MET: metabolic equivalent
MVPA: moderate-to-vigorous physical activity
PA: physical activity
SD: standard deviation
TV: television

## References

[1] Dunstan DW, Howard B, Healy GN, Owen N (2012). Too much sitting – a health hazard. Diabetes Res Clin Pract.

[2] Vandelanotte C, Sugiyama T, Gardiner P, Owen N (2009). Associations of leisure-time internet and computer use with overweight and obesity, physical activity and sedentary behaviors: cross-sectional study. J Med Internet Res.

[3] Healy GN, Wijndaele K, Dunstan DW, Shaw JE, Salmon J, Zimmet PZ (2008). Objectively measured sedentary time, physical activity, and metabolic risk: the Australian Diabetes, Obesity and Lifestyle Study (AusDiab). Diabetes Care.

[4] van der Ploeg HP, Chey T, Korda RJ, Banks E, Bauman A (2012). Sitting time and all-cause mortality risk in 222 497 Australian adults. Arch Intern Med.

[5] Oliver M, Schluter PJ, Healy GN, el Tautolo S, Schofield G, Rush E (2013). Associations between breaks in sedentary time and body size in Pacific mothers and their children: findings from the Pacific Islands Families Study. J Phys Act Health.

[6] Latouche C, Jowett JB, Carey AL, Bertovic DA, Owen N, Dunstan DW (2013). Effects of breaking up prolonged sitting on skeletal muscle gene expression. J Appl Physiol.

[7] Dunstan DW, Kingwell BA, Larsen R, Healy GN, Cerin E, Hamilton MT (2012). Breaking up prolonged sitting reduces postprandial glucose and insulin responses. Diabetes Care.

[8] Healy GN, Clark BK, Winkler EA, Gardiner PA, Brown WJ, Matthews CE (2011). Measurement of adults’ sedentary time in population-based studies. Am J Prev Med.

[9] Church TS, Thomas DM, Tudor-Locke C, Katzmarzyk PT, Earnest CP, Rodarte RQ (2011). Trends over 5 decades in U.S. occupation-related physical activity and their associations with obesity. PLoS One.

[10] Aadahl M, Andreasen AH, Hammer-Helmich L, Buhelt L, Jorgensen T, Glumer C (2013). Recent temporal trends in sleep duration, domain-specific sedentary behaviour and physical activity. A survey among 25–79-year-old Danish adults. Scand J Public Health.

[11] Alkhajah TA, Reeves MM, Eakin EG, Winkler EA, Owen N, Healy GN (2012). Sit-stand workstations: a pilot intervention to reduce office sitting time. Am J Prev Med.

[12] Adams MM, Davis PG, Gill DL (2013). A hybrid online intervention for reducing sedentary behavior in obese women. Front Public Health.

[13] Cooley D, Pedersen S, Mainsbridge C. Assessment of the impact of a workplace intervention to reduce prolonged occupational sitting time. Qual Health Res. 2013. doi:10.1177/1049732313513503. 10.1177/1049732313513503 24231074

[14] Carr LJ, Karvinen K, Peavler M, Smith R, Cangelosi K (2013). Multicomponent intervention to reduce daily sedentary time: a randomised controlled trial. BMJ Open.

[15] Healy GN, Eakin EG, Lamontagne AD, Owen N, Winkler EA, Wiesner G (2013). Reducing sitting time in office workers: short-term efficacy of a multicomponent intervention. Prev Med.

[16] Evans RE, Fawole HO, Sheriff SA, Dall PM, Grant PM, Ryan CG (2012). Point-of-choice prompts to reduce sitting time at work: a randomized trial. Am J Prev Med.

[17] Pedersen SJ, Cooley PD, Mainsbridge C (2014). An e-health intervention designed to increase workday energy expenditure by reducing prolonged occupational sitting habits. Work (Reading, MA).

[18] Swartz AM, Rote AE, Welch WA, Maeda H, Hart TL, Cho YI (2014). Prompts to disrupt sitting time and increase physical activity at work, 2011–2012. Prev Chronic Dis.

[19] Clemes SA, Patel R, Mahon C, Griffiths PL. Sitting time and step counts in office workers. Occup Med (Lond). 2014. doi:10.1093/occmed/kqt164. 10.1093/occmed/kqt164 24477502

[20] Chau JY, der Ploeg HP, van Uffelen JG, Wong J, Riphagen I, Healy GN (2010). Are workplace interventions to reduce sitting effective? A systematic review. Prev Med.

[21] Freak-Poli RL, Cumpston M, Peeters A, Clemes SA (2013). Workplace pedometer interventions for increasing physical activity. Cochrane Database Syst Rev.

[22] Miller R, Brown W (2004). Steps and sitting in a working population. Int J Behav Med.

[23] Tudor-Locke C, Burton NW, Brown WJ (2009). Leisure-time physical activity and occupational sitting: associations with steps/day and BMI in 54–59 year old Australian women. Prev Med.

[24] Shrestha N, Ijaz S, Kukkonen-Harjula KT, Kumar S, Nwankwo CP (2015). Workplace interventions for reducing sitting at work. Cochrane Database Syst Rev.

[25] Verweij LM, Proper KI, Weel AN, Hulshof CT, van Mechelen W (2012). The application of an occupational health guideline reduces sedentary behaviour and increases fruit intake at work: results from an RCT. Occup Environ Med.

[26] Ghoorah K, Campbell P, Kent A, Maznyczka A, Kunadian V. Obesity and cardiovascular outcomes: a review. Eur Heart J Acute Cardiovasc Care. 2014. doi:10.1177/2048872614523349. 10.1177/2048872614523349 24526749

[27] Bergouignan A, Momken I, Schoeller DA, Normand S, Zahariev A, Lescure B (2010). Regulation of energy balance during long-term physical inactivity induced by bed rest with and without exercise training. J Clin Endocrinol Metab.

[28] World Medical Association (2008). Declaration of Helsinki – ethical principles for medical research involving human subjects. WMJ.

[29] Lohman TG, Roche AS, Martorell R (1988). Anthropometric standardization reference manual.

[30] Godfrey A, Culhane KM, Lyons GM (2007). Comparison of the performance of the ActivPAL Professional physical activity logger to a discrete accelerometer-based activity monitor. Med Eng Phys.

[31] Kozey-Keadle S, Libertine A, Lyden K, Staudenmayer J, Freedson PS (2011). Validation of wearable monitors for assessing sedentary behavior. Med Sci Sports Exerc.

[32] Dowd KP, Harrington DM, Bourke AK, Nelson J, Donnelly AE (2012). The measurement of sedentary patterns and behaviors using the ActivPAL Professional physical activity monitor. Physiol Meas.

[33] Trost SG, McIver KL, Pate RR (2005). Conducting accelerometer-based activity assessments in field-based research. Med Sci Sports Exerc.

[34] Ward DS, Evenson KR, Vaughn A, Rodgers AB, Troiano RP (2005). Accelerometer use in physical activity: best practices and research recommendations. Med Sci Sports Exerc.

[35] Colley R, Connor Gorber S, Tremblay MS (2010). Quality control and data reduction procedures for accelerometry-derived measures of physical activity. Health Rep.

[36] Troiano RP, Berrigan D, Dodd KW, Masse LC, Tilert T, McDowell M (2008). Physical activity in the United States measured by accelerometer. Med Sci Sports Exerc.

[37] Barwais FA, Cuddihy TF (2015). Empowering sedentary adults to reduce sedentary behavior and increase physical activity levels and energy expenditure: a pilot study. Int J Environ Res Public Health.

[38] Grant PM, Ryan CG, Tigbe WW, Granat MH (2006). The validation of a novel activity monitor in the measurement of posture and motion during everyday activities. Br J Sports Med.

[39] Aadahl M, Linneberg A, Moller TC, Rosenorn S, Dunstan DW, Witte DR (2014). Motivational counseling to reduce sitting time: a community-based randomized controlled trial in adults. Am J Prev Med.

[40] Smith L, Hamer M, Ucci M, Marmot A, Gardner B, Sawyer A (2015). Weekday and weekend patterns of objectively measured sitting, standing, and stepping in a sample of office-based workers: the active buildings study. BMC Public Health.

[41] Schuna JM, Swift DL, Hendrick CA, Duet MT, Johnson WD, Martin CK (2014). Evaluation of a workplace treadmill desk intervention: a randomized controlled trial. J Occup Environ Med.

[42] Otten JJ, Jones KE, Littenberg B, Harvey-Berino J (2009). Effects of television viewing reduction on energy intake and expenditure in overweight and obese adults: a randomized controlled trial. Arch Intern Med.

[43] Harrington DM, Welk GJ, Donnelly AE (2011). Validation of MET estimates and step measurement using the ActivPAL physical activity logger. J Sports Sci.

[44] Neuhaus M, Healy GN, Dunstan DW, Owen N, Eakin EG (2014). Workplace sitting and height-adjustable workstations: a randomized controlled trial. Am J Prev Med.

[45] Pesola AJ, Laukkanen A, Haakana P, Havu M, Saakslahti A, Sipila S, et al. Muscle inactivity and activity patterns after sedentary-time targeted RCT. Med Sci Sports Exerc. 2014. doi:10.1249/mss.0000000000000335. 10.1249/MSS.0000000000000335 24674974

[46] Parry S, Straker L, Gilson ND, Smith AJ (2013). Participatory workplace interventions can reduce sedentary time for office workers – a randomised controlled trial. PLoS One.

