# Supplementary material for: Randomized controlled pilot of an intervention to reduce and break-up overweight/obese adults’ overall sitting-time
Source: Trials. 2015 Nov 2;16:490. doi: 10.1186/s13063-015-1015-4 (PMC4631103; doi:10.1186/s13063-015-1015-4)
Supplement: Additional file 2: — CONSORT 2010 flow diagram. (DOC 48 kb) [file 13063_2015_1015_MOESM2_ESM.doc]

**
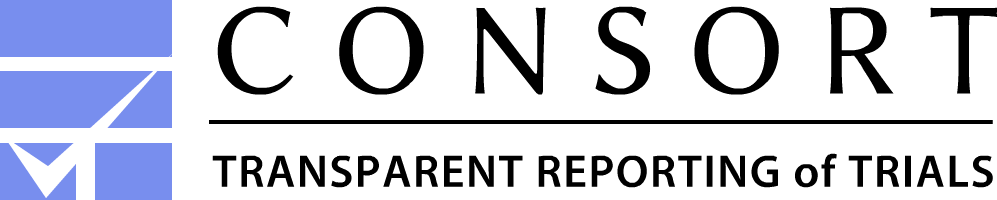
**

**CONSORT 2010 Flow Diagram**

**Allocation**

**Analysis**

**Follow-Up**

**Enrollment**

Assessed for eligibility (n=30)

Excluded (n=20)

♦  Not meeting inclusion criteria (n=20)

♦  Declined to participate (n=0)

♦  Other reasons (n=0)

Analysed (n=5)
♦ Excluded from analysis (give reasons) (n=0)

Lost to follow-up (give reasons) (n=0)

Discontinued intervention (give reasons) (n=0)

Allocated to intervention (n=5)

♦ Received allocated intervention (n=5)

♦ Did not receive allocated intervention (give reasons) (n=0)

Lost to follow-up (give reasons) (n=0)

Discontinued intervention (give reasons) (n=0)

Allocated to intervention (n=5)

♦ Received allocated intervention (n=5)

♦ Did not receive allocated intervention (give reasons) (n=0)

Analysed (n=5)
♦ Excluded from analysis (give reasons) (n=0)

Randomized (n=10)
